# Commercial Edible Seaweeds: Characterization by Mineral Content and Fatty Acid Composition

**DOI:** 10.3390/foods15183219

**Published:** 2026-09-11

**Authors:** Vincenzo Nava, Angela Giorgia Potortì, Ambrogina Albergamo, Roberto Sturniolo, Irene Maria Spanò, Giuseppa Di Bella

**Affiliations:** 1 Department of Biomedical, Dental and Morphological and Functional Imaging Sciences (BIOMORF), University of Messina, Viale Palatucci 13, 98168 Messina, Italy; vnava@unime.it (V.N.); agpotorti@unime.it (A.G.P.); roberto.sturniolo@studenti.unime.it (R.S.); irene.spano@studenti.unime.it (I.M.S.); gdibella@unime.it (G.D.B.); 2 Department of Chemical, Biological, Pharmaceutical and Environmental Sciences (CHIBIOFARAM), University of Messina, 98166 Messina, Italy

**Keywords:** seaweeds, mineral content, fatty acid composition, food safety, food quality

## Abstract

In recent years, seaweeds have attracted increasing scientific and commercial interest thanks to their protein, lipid and mineral content, and the presence of bioactive compounds that can have a positive impact on health. In light of this, seaweed is an innovative plant food source. This study characterizes commercial edible seaweeds (dulse, wakame, kombu, arame, nori, spirulina), evaluating their mineral composition, with particular attention to toxic or potentially toxic elements, and their fatty acid profile. The results revealed significant differences between species, which are probably related to taxonomic and environmental factors. Brown algae exhibited the highest levels of macroelements, particularly K and Na, which is consistent with their alginate-rich cell walls. Other species exhibited significant concentrations of Fe and Zn, while Pb and Hg remained below regulatory limits. However, nearly all samples exceeded the recommended limits of 0.5 mg/kg for Cd and 3 mg/kg for total As, as defined by the Centre d’Étude et de Valorisation des Algues (CEVA). Additionally, kombu samples had an extremely high iodine content, exceeding the upper CEVA-recommended limit of 2000 mg/kg. These values, therefore, require targeted risk assessments and consumer guidance. The fatty acid profile also appeared to vary by taxa. Red algae, especially nori, were rich in EPA, while brown species showed heterogeneous distributions of SFA, MUFA, and PUFA. Spirulina displayed a distinctive SFA- and n-6 PUFA-rich profile. Overall, the results confirm the need for careful management of iodine, Cd and As levels to ensure that these seaweeds can be used safely as food supplements and to promote their use in innovative food products.

## 1. Introduction

Although seaweeds have been consumed since ancient times, they have not yet gained widespread recognition in modern diets. This means that they are a largely underutilized plant-based food source. Despite their high potential in various fields, including nutrition, the use of seaweed in human diets has followed different cultural paths depending on the geographical region. In East Asia, in particular, seaweed has been widely consumed for thousands of years and remains a fundamental component of the traditional diet to this day [1]. Today, Japan, South Korea and China are among the world’s leading producers and consumers of edible seaweed. Products such as nori, kombu and wakame are an integral part of their daily diet.

By contrast, Western countries have not developed a similar tradition or widespread appreciation of using them as food. The ancient Greek and Roman civilizations, for instance, held the consumption of algae in low regard, and their use as a food source was largely abandoned in Europe and North America over the last century.

However, there is a growing interest in using edible seaweed in functional foods and gastronomy [2]. The growing importance of these matrices is related to their nutritional characteristics.

In fact, seaweed contributes to the dietary intake of minerals (e.g., Mg, Fe, and Zn), polysaccharides, proteins, fibers, polyunsaturated fatty acids and vitamins [3]. However, the chemical composition of algae varies depending on the type. Seaweeds can be classified into brown (*Phaeophyceae*), green (*Chlorophyceae*) and red (*Rhodophyceae*) algae, also known as macroalgae, depending on the characteristics of their pigments. *Rhodophyceae* may also exhibit shades of red, such as brownish red and purple. Microalgae (*Cyanophyceae*), also known as blue–green algae, are often unicellular and microscopic in size [4].

Thanks to their rich and varied nutritional profile, they are considered a viable alternative to conventional protein sources [5]. Indeed, variable protein content exists not only between microalgae and macroalgae, but also within individual classes (range: 7–75%) [4]. Blue–green microalgae can have a protein content of 55–74%, whereas some red and green macroalgae have a content of 26–29%. Other microalgal species, however, have lower protein levels, typically ranging from 16% to 32%. Although macroalgae are generally less protein-rich than microalgae [6,7], their protein content can vary depending on the growth environment and harvest location.

There are also marked differences in terms of lipids. Microalgae generally have a higher content, ranging from 2% to 7%, while the average content of macroalgae is around 2%. Nevertheless, both microalgae and macroalgae boast an intriguing lipid profile, characterized by a high proportion of unsaturated fatty acids, including EPA and DHA—molecules renowned for their positive impact on cardiovascular and neurological health [8,9,10].

Both micro- and macroalgae are a significant source of dietary fiber, vitamins (including vitamin B12, although its bioavailability may vary) and antioxidant pigments that protect the body against cardiovascular disease, particularly atherosclerosis [11].

Alongside this potential, it is also necessary to consider critical food safety issues. Algae can accumulate toxic and potentially toxic elements, such as arsenic (As), lead (Pb), mercury (Hg), and cadmium (Cd), as well as other environmental contaminants. The close link between potential contamination by toxic and potentially toxic substances and habitat and ecology is no coincidence [12]. However, there may also be other potential risks to humans associated with organic (e.g., polychlorinated biphenyls and dioxins) and microbiological (e.g., *Salmonella* spp., *Bacillus* spp.) contamination [13,14]. Therefore, a rigorous assessment of the origin and quality of algae-based products is essential, particularly in relation to current regulatory limits.

Interest in edible seaweed is also steadily growing in Europe, but there are still no harmonized European-level regulations establishing maximum limits for inorganic elements in seaweed and seaweed-derived products. The only exception is Commission Regulation (EU) 2023/915, which sets maximum levels for Cd, Pb, and Hg only in algae-based dietary supplements. This regulatory gap clearly challenges consumer protection, as seaweed has a marked ability to accumulate toxic and potentially toxic elements, such as As, Cd, Pb and Hg, in concentrations that depend heavily on habitat, ecological conditions and the area in which it is harvested.

The lack of shared reference values at the European level means that consumer protection is inconsistent and, in some cases, inadequate. In a market where seaweed is playing an increasingly significant role as both a culinary ingredient and a component of functional foods, the absence of harmonized limits exposes consumers to potential toxicological risks, particularly with regard to regular or habitual consumption.

In this context, the originality of the present study lies in the integrated investigation of different dimensions of nutritional quality and food safety in the same commercial products. Indeed, previous works have typically investigated mineral content and fatty acid composition in edible seaweeds separately. In contrast, this study assesses the fatty acid composition and nutritional indices of lipids alongside macroelements, microelements, and toxic inorganic contaminants, verifying their compliance with the reference limits set out in international regulations and by the Centre d’Étude et de Valorisation des Algues (CEVA). The latter organization is a key technical and scientific reference point for the algae industry. Moreover, the study incorporates a toxicological risk assessment based on realistic consumption scenarios, including both Asian dietary habits and a conservative European intake model. This combined evaluation provides a comprehensive and practical perspective that is directly relevant to food producers and regulatory authorities, and, not least, consumers.

In light of this, the aim of this manuscript is to evaluate the nutritional value of the main commercially available species of edible seaweed in terms of mineral profile and fatty acid (FA) composition. The study also aims to analyze the presence of toxic and potentially toxic inorganic elements and verify their compliance with the current limits fixed by the available national regulations. Furthermore, the toxicological risk associated with consuming a standard serving of seaweed is estimated. In view of the increasing popularity of these products in the European market and the ongoing absence of harmonized legislation, this study seeks to emphasize not only the nutritional and toxicological relevance of monitoring edible seaweeds but also the importance of establishing shared reference limits for these xenobiotics, even in countries where seaweed consumption is not as widespread as it is in Asia.

## 2. Materials and Methods

### 2.1. Material and Reagents

For the study of the mineral profile, reagents [i.e., H_2_O_2_ (30%, *v*/*v*) and HNO_3_ (65%, *v*/*v*)] and ultrapure water were from J.T. Baker (Milan, Italy). Stock solutions of reference standards of macroelements (i.e., Na, Mg, K, Ca and P), trace elements (i.e., Fe, Zn, Cu, Mn, Se, Cr, Sr, Mo, I and Co) and (potentially) toxic microelements (i.e., Ni, Sn, Sb, As, Cd, Pb and Hg) were supplied by Fluka (Milan, Italy) at a concentration of 1000 mg/L in 2% HNO_3_, each. The certified reference material (CRM) NIST1570 a (spinach leaves) was purchased from the National Institute of Standards and Technology (NIST, Gaithersburg, MD, USA) and used for quality assurance of the analytical procedure. For the study of the FA composition, n-heptane (reagent-grade) was manufactured by J.T. Baker (Phillipsburg, NJ, USA). Commercial standards of fatty acid methyl esters (FAMEs, C4–C24) were provided by Sigma-Aldrich (St. Louis, MO, USA).

### 2.2. Description of the Samples

This study analyzed six different types of edible seaweed: *Palmaria palmata* (dulse), *Porphyra yezoensis* (nori), *Undaria pinnatifida* (wakame), *Eisenia bicyclis* (arame), *Laminaria ochroleuca* (kombu) and *Arthrospira platensis* (spirulina) (see Table 1). One sample of each type of seaweed was purchased online in March 2025 in dried form, preliminarily ground into a homogeneous powder, and stored in airtight containers away from light and humidity until analysis. Three analytical replicates were taken from each homogenized sample. Furthermore, the geographical origins of the analyzed algae varied and are listed in Table 1.

### 2.3. Mineralization of Samples and Determination of Inorganic Elements

Approximately 0.5 g of each analyzed algae sample was treated with 8 mL of HNO_3_ (69% *v*/*v*) and 2 mL of H_2_ O_2_ (30% *v*/*v*), and mineralization was carried out using an Ethos 1 microwave mineralizer (Milestone, Bergamo, Italy), according to Panebianco et al. (2021) [3]. The operating conditions were as follows: first step: 10 min at 1000 W with a gradual increase up to 200 °C; second step: 10 min at 1000 W while maintaining a constant temperature of 200 °C; and third step: 20 min cooling. After digestion, the mineralized samples were diluted with ultrapure water, filtered with a 0.45 μm syringe filter and stored at 4 °C until analysis. These operating conditions were those described in a previous article of ours. In addition to the samples, the solution blank and the CRM NIST1570 a underwent the same pretreatment process. All samples were prepared in triplicate. The concentration of the 21 analyzed inorganic elements (Ca, K, Na, Mg, P, Fe, Zn, Cu, Se, Mn, Cr, Sr, Mo, Ni, Sb, Sn, I, Co, Cd, As and Pb) was determined using inductively coupled plasma mass spectrometry (iCAP-Q ICP-MS, Thermo Scientific, Waltham, MA, USA) and inductively coupled plasma optical emission spectroscopy (ICP-OES ULTIMA 2, HORIBA, Kyoto, Japan). The instrumental conditions for the two instruments were the same as those described in our previous study [15]. However, Hg was determined using a direct mercury analyzer (DMA-80, Milestone, Bergamo, Italy). The operating conditions were the same as those reported by Ben Amar et al. [16], and triplicate analyses were conducted for each sample. All methods were validated for linearity, sensitivity, accuracy and precision. See the Supplementary Materials for specifications.

### 2.4. Extraction of the Lipid Fraction and Analysis of Its FA Profile

The total lipid content was determined using the AOAC 920.39 method [17]. Approximately 10 g of dried algae were subjected to Soxhlet extraction using n-heptane as the solvent for six hours. The lipid extract was then concentrated using a Heidolph Instruments GmbH & Co. rotary evaporator (Schwabach, Germany) under vacuum conditions, and the extraction yield was quantified gravimetrically. The lipid fraction obtained by Soxhlet extraction was used to characterize the fatty acid composition. Specifically, derivatization of the lipid extract with heptane and 2 M methanolic KOH was performed to synthesize fatty acid methyl esters (FAMEs) [18]. The mixture was left to stand until two phases formed. The resulting supernatant was then collected and injected into a gas chromatograph equipped with a split/splitless injector and a flame ionization detector (GC-FID, Dani Master GC, Dani Instrument, Milan, Italy). The operating conditions were as described by Lo Turco et al. [19].

Data acquisition and analysis were performed using Clarity Chromatography Software (version 4.0.2). The fatty acid methyl esters (FAMEs) were identified by comparing their retention times with those of the reference standards, and the relative percentages of the individual compounds were calculated based on the total area of the chromatogram. To ensure reproducibility of the results, the analyses were performed in triplicate for each sample. The assessment of the health and nutritional potential of the lipids in seaweeds was conducted using the main nutritional indices, such as the atherogenicity index (AI) and the thrombogenicity index (TI). The AI is calculated by adding together the amounts of the main saturated fatty acids and dividing this sum by the total amount of the main classes of unsaturated fatty acids, following the formula reported by Nava et al. [20]:

AI = [C12:0 + (4 × C14:0) + C16:0]/[Σ n-6 PUFA + Σ MUFA + Σ n-3 PUFA]

Conversely, the thrombogenic index (TI) indicates the tendency to generate blood clots. This is ascertained by calculating the proportion of pro-thrombogenic (SFA) to anti-thrombogenic (MUFA, n-6 PUFA and n-3 PUFA) fatty acids [20]:

TI = [C14:0 + C16:0 + C18:0]/[0.5 × Σ n-6 PUFA + 0.5 × Σ MUFA + 3 × Σ n-3 PUFA + (n-3 PUFA/n-6 PUFA)]

### 2.5. Uptake of Inorganic Elements

In this study, the toxicological risk to consumer health resulting from consuming an amount of seaweed was also assessed. The recommended daily allowances (RDAs) and the daily/weekly doses or the lower limit of the 95% confidence interval of the reference dose (BMDL01), as specified by various European regulatory bodies [21,22,23,24,25,26,27,28,29], were used to compare the uptake of essential elements and toxic and potentially toxic elements. For the latter, intake was calculated based on an average body weight of 60 kg.

Due to variations in global seaweed consumption, it is not possible to establish a single standard amount applicable to all populations. For this reason, the following study adopted the approach proposed by the National Food Institute [30]. They assessed the potential health risks associated with consuming unprocessed marine seaweed and estimated average intake levels according to dietary habits. Specifically, the following values were considered for the groups that consume the highest amounts of this food group: 5.2 g/adult/day for the Chinese population; 4 g/adult/day for the Japanese population; and 8.5 g/adult/day for the South Korean population. However, the lack of consolidated data on seaweed consumption among the European population made it necessary to adopt a conservative assumption for the purposes of risk assessment. It was, therefore, assumed that a single serving of 5 g would be consumed once a week, representing occasional but plausible consumption in the European context.

### 2.6. Statistical Analysis

For the statistical analysis, GraphPad Prism Version 10.3.1 (GraphPad Software, Inc., Boston, MA, USA) was used. A one-way ANOVA test with a post hoc Tukey’s HSD test was used to evaluate the significant differences between seaweed types. We set the level of statistical significance at *p* < 0.05.

## 3. Results and Discussions

### 3.1. Macro-Elements

Table 2 shows the average content of five macro-elements (Ca, K, Na, Mg and P) of different types of seaweeds analyzed, enabling a detailed assessment of each sample. The Table also presents the average concentrations of the main algae types—namely red macroalgae, brown macroalgae and green microalgae—providing a comprehensive overview useful for comparing average levels across different taxonomic groups.

In general, all the analyzed seaweeds were an excellent source of macronutrients. However, the levels of Ca, K, Na, Mg and P varied depending on the type of seaweed (*p*-values < 0.05), as reported also in the literature [31]. In particular, kombu had the highest Ca (10,253.60 ± 51.60 mg/kg), K (46,360.12 ± 548.84 mg/kg) and Na (49,419.96 ± 1035.35 mg/kg) levels. Arame had the highest Mg concentration (17,496.40 ± 157.80 mg/kg), and spirulina had the highest P content (8912.58 ± 117.20 mg/kg). Compared to red algae, brown macroalgae had the highest average content of Ca (8803.18 ± 1688.03 mg/kg), Na (27,052.75 ± 20,013.30 mg/kg) and Mg (13,822.77 ± 3464.25 mg/kg). This trend corroborates the results of several previous studies [31,32,33]. The only macroelement in which red algae exceeded brown algae was in terms of average K content (28,026.49 ± 3469.07 mg/kg vs. 23,438.40 ± 21,412.61 mg/kg). In general, the higher concentration of macronutrients found in brown algae may be due to the presence of polysaccharides and alginates in their cell walls. These substances have a particular affinity for Ca, Mg, Na and K salts [34]. Spirulina, a blue–green microalgae, has higher P amounts than other types of algae, with an average content of 8912.58 ± 117.20 mg/kg. These levels are consistent with those reported by Janda-Milczarek et al. for spirulina powder samples (mean: 11,225.16 ± 2069.442 mg/kg) [35]. The variability in the composition of this class of minerals could be considered a strength, as it enables the selection of the most suitable species for specific nutritional requirements. This confirms the versatility and high value of seaweed as a functional food, which is superior to traditional vegetables in terms of macronutrients [36]. This is important because these macronutrients play a crucial role in the human body: Ca is essential in bone health and various metabolic processes [37]; K in different pathologic states (e.g., cardiovascular system, kidneys and musculoskeletal health) [38]; Mg plays a key role in maintaining cardiovascular health and in immune response [39]; P involves in a wide variety of essential cellular functions [40]; and Na is required for many physiological processes, but high sodium intake can lead to negative effects on blood pressure and, consequently, to cardiovascular disease [41].

### 3.2. Trace Elements

Table 3 shows the average content of 11 trace elements (Fe, Zn, Cu, Se, Mn, Cr, Sr, Mo, I, Co). Similarly, the average concentrations of the main types of algae—namely red macroalgae, brown macroalgae and green microalgae—are reported.

The elemental analysis of the examined edible algae samples revealed a variable trace element profile (*p*-values <0.05 for all trace elements; see Table 3). In general, good concentrations of Fe, I, Zn, Mn, Cu, Sr and Cr were observed in all the samples analyzed (see Table 3). Fe is one of the most abundant trace elements. The highest levels were found in spirulina (554.96 ± 4.17 mg/kg) and dulse (407.07 ± 5.62 mg/kg), followed by kombu (301.64 ± 3.35 mg/kg), arame (255.25 ± 6.63 mg/kg), wakame (159.30 ± 7.55 mg/kg) and nori (130.25 ± 3.53 mg/kg). Additionally, blue–green microalgae exhibited the highest average Fe amounts (554.96 ± 4.17 mg/kg), surpassing the other two classes of macroalgae: red macroalgae (268.66 ± 195.75 mg/kg) and brown macroalgae (238.73 ± 72.59 mg/kg). This is consistent with previous studies showing that spirulina is an excellent Fe source. However, it is important to consider which form is more bioavailable: Fe^2+^ or Fe^3+^. For example, Masten Rutar et al. [42] analyzed the Fe content of spirulina-based products available in Slovenia. They found that Fe concentrations ranged from 370 to 3480 mg/kg and that 82–92% of this metal was present as Fe^3+^ rather than Fe^2+^.

The I content is particularly noteworthy. The EU published Recommendation (EU) 2018/464 on the monitoring of I and other elements (such as As, Cd, Pb, and Hg) in seaweed species used for food and feed [43]. With values of 3125.61 ± 54.48 mg/kg, kombu seaweed showed an extremely high I content compared to other seaweeds. On average, brown macroalgae had significantly higher I levels (1137.52 ± 1722.93 mg/kg) than red algae (112.60 ± 106.34 mg/kg) and blue–green microalgae (10.56 ± 0.95 mg/kg). The variability in this element may be due to several factors influencing the accumulation of this element in algae, including water salinity, temperature, depth and thallus age [44]. According to CEVA recommendations and guidelines, the maximum permitted I content in seaweed intended for human consumption is 2000 mg/kg of dry weight [45]. The results of this study show that almost all of the analyzed samples fell well within this limit, except for kombu seaweed. The element iodine is an essential nutrient for synthesizing thyroid hormones (TH). However, the relationship between iodine intake and thyroid disorders is not straightforward—it actually follows a U-shaped curve. This means that both insufficient and excessive iodine intake can lead to thyroid dysfunction [46]. Consequently, it is recommended to exercise caution and meticulously monitor your intake of this element, as excessive consumption can elicit deleterious effects in certain individuals, including hyperthyroidism, hypothyroidism, goiter, or thyroid autoimmunity [47].

High concentrations of Zn were also observed. The highest values were found in nori (38.89 ± 1.45 mg/kg) and the lowest in spirulina (14.08 ± 1.30 mg/kg). Red macroalgae had the highest Zn levels (34.46 ± 6.27 mg/kg) in this case, followed by brown macroalgae (26.46 ± 9.85 mg/kg) and blue–green microalgae (14.08 ± 1.30 mg/kg). The Zn content of arame, kombu, nori and wakame seaweeds was compared with that reported by Besada et al. (2009) for samples of the same seaweed types purchased in Spain [48]. The concentrations were almost always comparable, except for wakame, for which the concentration was higher in our study (37.66 ± 1.45 vs. 8.25–26.6 mg/kg). Furthermore, the Zn content of red macroalgae was higher than that reported by Khandaker et al. (2021) for the red alga *Eucheuma cottoni* collected from various regions of Malaysia (mean value: 18.1 mg/kg) [49]. Regarding brown macroalgae, our average Zn content in wakame seaweed was comparable to that reported by Paz et al. for commercial European wakame (37.66 ± 1.45 mg/kg vs. 40.3 ± 32 mg/kg), but higher than that of Asian wakame (18.1 ± 4.3 mg/kg) [50]. Differences in the content of this element have also been observed in kombu seaweed (22.57 ± 1.51 mg/kg vs. 10.3 ± 7.1 mg/kg) [50]. This demonstrates that Zn can vary not only in relation to the type/class of algae but also in relation to the different geographic origin.

The analyzed seaweeds also proved to be an important source of Mn, with concentrations ranging from 7.03 ± 0.56 mg/kg in wakame to 33.14 ± 1.61 mg/kg in arame. Our concentrations of this element in wakame and kombu were higher than those reported by Paz et al. (wakame: 7.03 ± 0.56 vs. 2.71 ± 1.1/5.64 ± 0.70 mg/kg; kombu: 17.22 ± 1.66 vs. 8.61 ± 13 mg/kg) [50]. By contrast, the average Mn content determined for the red macroalgae nori was lower than the range reported by Rodrigues Albuquerque et al. (18.93 ± 1.05 mg/kg vs. 14.6 ± 0.8–393 ± 10 mg/kg) [51]. Furthermore, the average Mn content of brown algae was lower than that of red algae and blue–green microalgae (see Table 3). This finding is consistent with previous studies [34]. The high concentrations of Mn found in our algae samples are significant, given that it is an essential mineral involved in many bodily functions, such as the metabolism of proteins, lipids and carbohydrates, the proper functioning of the immune system, and the regulation of blood glucose levels [52]. However, excess Mn can cause problems, as it appears to be associated with various neuropsychiatric disorders [50,52].

Cu ranged from 2.11 ± 0.13 mg/kg in kombu to 7.92 ± 0.19 mg/kg in nori. Similar results were reported in a study by Miedico et al. on three species of edible seaweed: *Porphyra, Laminaria* and *Undaria.* As in our study, *Porphyra* species exhibited higher average concentrations (16.9 ± 7.6 mg/kg) than the other two species [53]. Furthermore, our study found that the Cu content of red macroalgae (6.22 ± 2.41 mg/kg) was higher than that of brown (3.22 ± 1.59 mg/kg) and blue–green microalgae (3.16 ± 0.16 mg/kg). These results are consistent with those reported by Rubio et al. in their study of edible algae from Asian and European regions [54]. However, the copper content can also vary depending on the type of alga, even within the same taxonomic class. For instance, the Cu content of the red macroalgae species analyzed in this study was higher than that reported by Ajik et al. for *Solieria robusta* (1.55 ± 0.02 mg/kg) [55]. This demonstrates that different types of algae accumulate these inorganic elements to varying degrees. Although this research found a moderate amount of copper in the studied algae, it is important to monitor copper consumption and absorption, as excessive intake could damage the liver [50].

High concentrations and considerable variability were observed for Sr. Wakame had the highest content (799.71 ± 3.93 mg/kg) in this case, while dulse had the lowest (25.82 ± 1.94 mg/kg). On average, brown macroalgae had significantly higher content than red algae and blue–green microalgae (400.29 ± 371.05 mg/kg vs. 29.75 ± 5.56 mg/kg vs. 46.03 ± 2.82 mg/kg, respectively). However, the literature also reports high concentrations of Sr. For instance, Siddique et al. reported strontium concentrations ranging from 1067.53 ± 6.21 mg/kg in the red alga *Gelidium pusillum* to 2838.86 ± 12.16 mg/kg in the brown alga *Padina tetrastromatica*, which confirms our order of abundance [56]. Similar results were observed in a study by Larrea-Marín et al., who found higher Sr content in *Laminaria* samples (1.14 ± 0.50 mg/g) than in Porphyra samples (0.13 ± 0.06 mg/g) [57]. By contrast, Pasumpon et al. obtained significantly lower values for various red and brown algae (range: 0.21 ± 0.01–1.38 ± 0.11 μg/g) [58]. The higher concentration of Sr in brown algae can be explained by their high alginate content. Alginates have ion-exchange properties that enhance Sr accumulation, particularly those rich in guluronic acid residues, which have a high affinity for Sr [59].

The average chromium content ranged from 0.57 ± 0.07 mg/kg in nori seaweed to 2.73 ± 0.10 mg/kg in kombu seaweed. Furthermore, the abundance order among the different taxonomic groups was as follows: brown macroalgae (2.10 ± 0.59 mg/kg) > blue–green microalgae (2.03 ± 0.08 mg/kg) > red macroalgae (1.24 ± 0.94 mg/kg). In general, our results were consistent with those of Smith et al. for edible seaweed purchased in New Zealand (range: 0.44 ± 0.23–2.61 ± 3.08 mg/kg) [60]. The same cannot be said when comparing the results with those of the Paz et al. study, in which the average Cr content ranged from 0.07 ± 0.12 to 0.50 ± 0.70 mg/kg. That is, it was lower than that found in our study [50].

Finally, the concentrations of Se, Mo and Co were all consistently below 1 mg/kg. Blue–green microalgae had the highest average Se content (0.96 ± 0.06 mg/kg), while red macroalgae had the highest Mo and Co contributions (0.44 ± 0.15 mg/kg and 0.31 ± 0.10 mg/kg, respectively).

### 3.3. Toxic and Potentially Toxic Elements

The analysis also involved the determination of toxic and potentially toxic elements: Cd, Pb, As, Hg, Ni, Sn, and Sb (Table 4). Similar to Table 2 and Table 3, the average concentrations of toxic and potentially toxic elements in the main types of algae—namely red macroalgae, brown macroalgae and green microalgae—are reported.

The concentrations of this class of inorganic elements may have been influenced by the geographic origin and species analyzed. In fact, *p*-values < 0.05 have been obtained for all toxic and potentially toxic elements (Table 4). However, it is difficult to determine whether the levels found in the analyzed algae are a cause for concern. In fact, despite the risk of contamination by toxic elements, marine algae are subject to minimal food safety regulations, particularly in Western countries [61]. Under European regulations, limits for marine algae exist only in relation to their use as an ingredient in animal feed [62]. However, there are no harmonized European reference limits for seaweed intended for human consumption. This highlights a significant gap in human food safety. For this reason, the present study refers to CEVA recommendations, a French organization that sets maximum levels for As, Cd, Pb, Hg, Sn and I in seaweed intended for human consumption (Table 5).

The average lead values ranged from 0.10 ± 0.02 mg/kg in nori to 0.82 ± 0.02 mg/kg in arame. Furthermore, the concentration of this toxic element was found to vary in the following order: brown macroalgae (0.43 ± 0.36 mg/kg) > red macroalgae (0.17 ± 0.10 mg/kg) > blue–green macroalgae (0.13 ± 0.01 mg/kg). The higher concentration of lead in brown algae may be related to their greater ability to absorb it due to their high polysaccharide content, with sulfate and carboxyl groups identified as effective metal-binding sites [61]. Generally, however, all the algae samples showed lead levels well below the French limit of 5 mg/kg on a dry weight basis.

The highest cadmium content was found in nori seaweed (1.06 ± 0.07 mg/kg), while the lowest was found in spirulina (0.22 ± 0.03 mg/kg). In general, red algae had a higher cadmium content than the other groups. The level of cadmium found in this study was similar to that reported by Chen et al. (2021) in samples of *Porphyra* and *Laminaria* purchased in China [63]. There is a European regulatory reference for this element, namely Regulation (EU) 2023/915. However, this sets a limit of 3 mg/kg for dietary supplements consisting of at least 80% dried seaweed [64]. For this reason, we decided to compare our concentrations with the French maximum limit of 0.5 mg/kg [45]. Consequently, more than half of the samples exceeded this threshold value. The varying concentration range observed for cadmium is related to the varying degree of pollution in the geographic area where the algae grow. Furthermore, it has generally been demonstrated that algae are good accumulators of cadmium [61].

The highest arsenic levels were found in kombu, ranging from 4.92 ± 0.39 mg/kg in spirulina to 45.63 ± 3.33 mg/kg in kombu. Furthermore, brown macroalgae had an average As content that was higher than that of red algae and blue–green microalgae. This trend was according to Chen et al. [63] and Todorov et al. [65]. The high concentrations of arsenic may generally be related to the role of algae as efficient filters and accumulators of the element [66]. Although European regulations set specific limits for inorganic arsenic in certain food categories, seaweed is not one of them. By contrast, French regulations stipulate a maximum limit of 3 mg/kg of inorganic arsenic in seaweed. Consequently, all of our samples showed total arsenic levels that exceeded this reference value. However, one important aspect must be taken into account. From a risk assessment perspective, the total arsenic content (i.e., the sum of inorganic and organic arsenic) in food has no toxicological significance [67]. Furthermore, although algae are known to contain high concentrations of total arsenic, most of this is in the form of organic species and the inorganic arsenic content is generally low [68]. Nevertheless, possible sources of contamination that could lead to an increase in this toxic species must be considered. Therefore, it is necessary to quantify the most toxic and dangerous form of arsenic, inorganic arsenic, in order to determine whether our results pose a risk to consumers. High concentrations of this toxic element can cause various health problems in consumers, such as liver, kidney, bladder and skin cancer [69].

The average Hg levels found in the analyzed samples were the lowest of all the toxic and potentially toxic elements tested. The concentrations ranged from 0.01 ± 0.00 mg/kg (dulse, wakame and nori) to 0.03 ± 0.01 mg/kg (kombu). This was consistent with the range reported in other studies [48,65]. Although there are no specific European limits for the ‘algae’ class, the “seafood” and “food supplements (including those prepared from seaweed)” categories can be referred to instead. EU Regulation 2023/915 sets reference limits ranging from 0.3 to 1.0 mg/kg for the first category, depending on the species, and 0.1 mg/kg for the second category. Furthermore, French CEVA regulations set a maximum Hg limit of 0.1 mg/kg [45]. Consequently, in both cases, our samples showed levels below the regulatory limits. However, although the concentrations are fortunately low, it would have been appropriate to evaluate the different forms of mercury present, given that the total mercury content has been determined. Inorganic forms of Hg have a short biological half-life and tend not to accumulate in the food chain. In contrast, methylmercury is absorbed almost entirely from the digestive tract at all trophic levels, has an enormous half-life and is subject to biomagnification [70].

Low concentrations of Sn and Sb were also found. For the first element, nori algae showed the lowest content (0.01 ± 0.00 mg/kg) and spirulina showed the highest (0.05 ± 0.01 mg/kg). However, the average content of Sn was also well within the French regulatory limit of 5 mg/kg. In the case of Sb, kombu had the lowest concentration (0.02 ± 0.00 mg/kg), while arame had the highest one (0.08 ± 0.01 mg/kg). There are no maximum reference limits for this element, but our results were comparable with those reported by Siddique et al. for red and brown algae collected from Saint Martin’s Island in the Bay of Bengal, Bangladesh [56].

The algae samples analyzed in this study also exhibited significant Ni content. The average concentration ranged from 0.51 ± 0.05 mg/kg in spirulina to 4.01 ± 0.11 mg/kg in dulse. Red macroalgae had the highest average content of this element among the categories investigated (2.27 ± 2.46 mg/kg). The results are comparable to those reported by Siddique et al. (mean range: 2.52 ± 0.06 to 8.89 ± 0.03 mg/kg) [56]. The French regulation does not report maximum reference values for this element. However, EU Regulation 2024/1987 sets maximum Ni levels in certain food products, including seaweed. Specifically, this regulation stipulates two maximum levels: 30 mg/kg for all seaweed except wakame, for which the maximum level is 40 mg/kg [71]. However, all our samples were well within these normative levels.

### 3.4. Fatty Acid (FA) Composition

The FA profiles reported in Table 6 reveal marked taxonomic differentiation among the analyzed seaweeds, confirming that lipid composition is strongly species-dependent and shaped by both phylogeny and environmental factors. Although the total lipid content of macroalgae is generally low, the qualitative distribution of fatty acids highlights their potential nutritional relevance.

Analysis of the lipid profiles of the algae species studied revealed differences in terms of their content of saturated (SFAs), monounsaturated (MUFAs) and polyunsaturated (PUFAs) fatty acids. Variability in fatty acid composition, shown by *p*-values < 0.05 (Table 6) in all fatty acids (except for C20:3 n-3), may be due to genetic differences between species and their different geographic origins, as well as other abiotic factors, such as light, salinity and nutrients [72].

For most samples, SFAs represented the highest average content, except for red macroalgae such as dulse and nori, which had a higher average contribution of PUFAs. However, kombu had the highest average SFA content (44.25 ± 1.22 g/100 g), while nori had the lowest one (32.53 ± 0.85 g/100 g). The most abundant SFAs in all samples were palmitic acid (C16:0 range: 25.17 ± 0.87–33.35 ± 1.24 g/100 g) and stearic acid (C18:0 range: 1.19 ± 0.05–6.64 ± 0.03 g/100 g). For C16:0, Campos et al. obtained higher percentages in *Palmaria palmata* and *Porphyra* spp. samples from the Portuguese market (62.1 ± 0.22% and 41.9 ± 4.46%, respectively) than in our own samples [73]. Jaworowska et al. also obtained consistent results for C16:0 in red (*Palmaria palmata* and *Porphyra* spp.) and brown (*Laminaria digitata* and *Undaria pinnatifida)* macroalgae [74]. For C18:0, the concentrations of our dulse seaweed were higher than Jaworowska et al. (5.14 ± 0.24 vs. 1.54 g/100 g) [74], but comparable to those reported by Campos et al. (5.14 ± 0.24 vs. 5.8 ± 0.28 g/100 g) [73]. However, in the case of nori seaweed, our results were comparable to those of the two studies [73,74]. Spirulina exhibited C16:0 and C18:0 concentrations of 33.35 ± 1.24 g/100 g and 6.64 ± 0.03 g/100 g, respectively. These results are consistent with those obtained by Dibeklioglu et al., who found concentration ranges of 18.02 ± 0.78–38.56 ± 2.10% for C16:0 and 2.33 ± 0.68–7.60 ± 0.56% for C18:0 [75]. Our study found values ranging from 25.54 ± 0.90 g/100 g to 32.10 ± 1.35 g/100 g for C16:0, and from 1.94 ± 0.72 g/100 g to 2.82 ± 0.15 g/100 g for C18:0, in brown algae. In some cases, these ranges were comparable to the results obtained by Berneira et al. for other types of brown macroalgae. In other cases, however, they were less so (C16:0 = 20.80 ± 0.69–26.42 ± 1.11 g/100 g; C18:0 = n.d.–1.93 ± 0.11 g/100 g) [76]. This shows that the percentages of individual fatty acids can vary even within the same taxonomic category, depending on various factors such as genetic and environmental variables, species, water temperature, nutrient availability, salinity and pH [76]. Good concentrations were also obtained for myristic acid (C14:0), with the average values ranging from 0.38 ± 0.02 g/100 g in nori to 4.52 ± 0.21 g/100 g in arame. Furthermore, in terms of taxonomic species, the order of abundance for C14:0 was brown macroalgae > red macroalgae > blue–green microalgae (see Table 6). The results obtained are comparable to those reported in the literature. For example, the spirulina content (0.48 ± 0.01 g/100 g) obtained in this study was comparable to the range reported by Tokuşoglu et al.: 0.41–0.46 g/100 g [77]. Regarding red algae, the C14:0 content of dulse seaweed was lower than the figure of 8.5 g/100 g reported by Razi Parjikolaei et al. [78] and by Jaworowska et al. (11.32 g/100 g) [74]. On the contrary, the percentage of nori seaweed was comparable to that of Jaworowska et al. (0.37 g/100 g) [74]. The same trend was observed for brown macroalgae, including wakame seaweed (3.90 ± 0.15 g/100 g vs. 3.58 g/100 g) [74]. Good variability was also observed for C20:0 and C24:0. The highest content of both was found in kombu seaweed (C20:0: 2.36 ± 0.08 g/100 g; C24:0: 3.62 ± 0.11 g/100 g), while the lowest content of both was found in nori seaweed (C20:0: 0.03 ± 0.00 g/100 g) and spirulina (C24:0: 0.03 ± 0.00 g/100 g). The other SFAs (C12:0, C15:0, C17:0, C22:0) are present in very small quantities (<1 g/100 g).

MUFAs almost always had the lowest percentage compared to SFAs and PUFAs. The only exception was arame (34.29 ± 0.98 g/100 g), where the order of abundance was SFAs, MUFAs, and PUFAs. Oleic acid (C18:1 n-9) was the most abundant MUFA in all analyzed samples, ranging from 15.37 ± 0.66 g/100 g in kombu to 27.68 ± 1.02 g/100 g in arame. The percentages of C18:1 n-9 found in our red algae were higher than those reported by Jaworowska et al. for *Palmaria palmata* (4.57 g/100 g) and *Porphyra* spp. (3.93 g/100 g) [74], and by Denis et al. for *Grateloupia turuturu* (6.2 ± 0.3 g/100 g) [79]. The wakame and arame seaweeds had higher concentrations of palmitoleic acid (C16:1 n-7) than the other species, at 5.24 ± 0.35 g/100 g and 5.33 ± 0.41 g/100 g, respectively. This pattern aligns with earlier research [74]. The other MUFAs (C16:1 n-9, C17:1, C18:1 n-7, C20:1 n-9, and C22:1 n-9) were almost always present in quantities of less than 1 g/100 g in all the analyzed products.

The PUFA content ranged from 28.93 ± 0.46 g/100 g in arame to 44.66 ± 1.16 g/100 g in nori. Red macroalgae had the highest content, followed by blue–green microalgae and brown macroalgae. The difference in PUFA content among the various algae analyzed could be attributed to several factors, including the taxonomic species and their growing environment. Indeed, algae cultivated in cold climates may accumulate more PUFAs [80]. Red macroalgae (dulse and nori) exhibited the most favorable PUFA profile from a nutritional standpoint. Both species were characterized by high PUFA levels, driven predominantly by eicosapentaenoic acid (EPA). In particular, nori showed the highest EPA percentage (33.65 ± 1.54 g/100 g) among all samples, followed by dulse (27.21 ± 0.01 g/100 g). This confirms the well-established role of red algae as natural sources of n-3 PUFA and suggests their potential use in functional foods aimed at increasing dietary EPA intake [81] PUFA levels in brown algae were markedly lower than in red algae, and EPA concentrations were modest (i.e., kombu: 8.45 ± 0.32 g/100 g; wakame: 5.81 ± 0.18 g/100 g; and arame: 3.44 ± 0.10 g/100 g). Significant quantities of arachidonic acid have been obtained from this class of macroalgae: (C20:4 n-6): wakame: 13.32 ± 0.75 g/100 g; kombu: 12.89 ± 0.60 g/100 g; and arame: 12.23 ± 0.58 g/100 g. These results are consistent with those of Jaworowska et al., who obtained mean C20:4 n-6 values of 13.74 g/100 g and 11.35 g/100 g for *Laminaria digitata* and *Undaria pinnatifida*, respectively [74]. These differences reflect species-specific metabolic pathways and environmental adaptations, and they suggest that brown algae may contribute more to n-6 intake than to n-3 PUFA supply. Notably, spirulina was the only species containing appreciable levels of docosahexaenoic acid (DHA) (C22:6 n-3 = 3.01 ± 0.03 g/100 g), a fatty acid present at low levels in macroalgae (range: 0.04 ± 0.00–0.07 ± 0.00 g/100 g). Generally, however, the results obtained for spirulina PUFAs were consistent with those reported in the literature [77]. In conclusion, the FA profiles of the analyzed seaweeds revealed clear taxonomic patterns, confirming that lipid composition is strongly species-dependent and shaped by both phylogenetic traits and ecological conditions. However, the qualitative distribution of fatty acids highlights their potential nutritional relevance, particularly in relation to long-chain PUFAs.

Table 6 also shows the atherogenicity and thrombogenicity index values. Determining these two parameters is essential for evaluating the health and nutritional potential of lipids in algae samples. In this regard, values of AI and TI lower than one are beneficial to human health. Conversely, higher values of these indices can lead to an increased risk of platelet aggregation and thrombus formation [20]. Fortunately, the analyzed samples in the present study always showed AI (range: 0.48 in nori–0.85 in kombu) and TI (range: 0.26 in nori–0.97 in spirulina) values lower than one. This is a good result in terms of safety and potential risks to consumers.

Nori seaweed stood out due to its high EPA content (33.65 ± 1.54 g/100 g), which was the highest among all the species analyzed. This value is nutritionally significant. EPA is a long-chain omega-3 fatty acid with proven cardioprotective and anti-inflammatory properties, and the ability to modulate endothelial function [82]. The concentration of EPA found in nori suggests its potential use as a source of this omega-3 fatty acid to boost dietary intake [83], for instance, in supplements and food formulations aimed at populations with low fish consumption. Furthermore, its high EPA content results in favorable atherogenicity (AI = 0.48) and thrombogenicity (TI = 0.26) indices, both of which are below 1, thereby reinforcing the health profile of nori seaweed.

The lipid profile of spirulina is distinctly different from that of macroalgae: it is rich in saturated fatty acids (SFAs) (40.87%) and n-6 fatty acids, particularly C18:2 n-6 (19.05%) and C18:3 n-6 (8.81%). While this composition is consistent with the species’ cyanobacterial nature, it has more complex nutritional implications. A high level of n-6 fatty acids, if not balanced by an adequate intake of n-3, can promote the synthesis of pro-inflammatory eicosanoids [84]. However, the presence of gamma-linolenic acid (GLA), which is known for its anti-inflammatory properties, and the unique presence of DHA (3.01 g/100 g), which is absent in the other species, give spirulina a distinctive functional profile. Although its TI value (0.97) is below 1, it is the highest among the analyzed species, reflecting an unfavourable balance between pro- and anti-thrombogenic fatty acids.

Table 6 also presents the omega-3/omega-6 ratio, a parameter that is widely used to evaluate the nutritional quality of lipids. Nori and dulse have high ratios of omega-3 to omega-6, which is consistent with their high content of eicosapentaenoic acid (EPA) and low levels of omega-6. This profile is considered optimal for cardiovascular health. Spirulina shows the least favorable ratio, with a predominance of n-6 despite the presence of DHA.

Overall, the results confirm that red macroalgae (nori and dulse) are the most promising sources of long-chain omega-3 fatty acids for use in functional foods and supplements. Brown macroalgae (wakame, arame and kombu) display more variable profiles and a higher n-6 contribution, whereas spirulina presents a unique lipid profile characterized by high levels of SFAs and n-6, with nutritional implications that call for more targeted use.

### 3.5. Elements Uptake

Given the wide and varied consumption of algae worldwide, this study aimed to evaluate the toxicological risk to consumer health by considering four different population groups characterized by their varying levels of algae consumption: 5.2 g/adult/day for the Chinese population; 4 g/adult/day for the Japanese population; 8.5 g/adult/day for the South Korean population; and 5.0 g/adult/week (0.7 g/adult/day) for the European population. The following RDAs were used as a reference for essential macronutrients and micronutrients: Ca (800 mg/day), Cr (0.040 mg/day), Cu (1 mg/day), Fe (14 mg/day), K (2000 mg/day), Na (1500 mg/day), Mg (375 mg/day), Mn (2 mg/day), Mo (0.050 mg/day), P (700 mg/day), Se (0.055 mg/day), Zn (10 mg/day), and I (0.15 mg/day [85]). For toxic and potentially toxic elements, the references TDI, TWI and BMDL01 were As (0.3 µg/kg_b.w._/day), Hg (4 µg/kg_b.w._/day), Ni (22 µg/kg_b.w._/day) and Pb (0.5 µg/kg_b.w._/day), Cd (2.5 µg/kg_b.w._/week), Sb (6 µg/kg_b.w./_week), and Co (1.6 µg/kg_b.w._/week). The intake percentage was calculated based on an average weight of 60 kg. Table 7 presents the results obtained from calculating the uptake of each inorganic element based on the varying quantities of algae consumed by the studied populations. The assessment of inorganic element absorption (see Table 7) reveals a clear discrepancy between the nutritional value of essential minerals and the toxicological impact of certain xenobiotics. The macronutrient absorption assessment revealed that the nutritional contribution of Ca, K, Na, Mg and P strongly depends on species and consumption habits. While all the algae analyzed were found to be an excellent source of macronutrients, the actual dietary impact on European consumers is modest due to the low assumed intake of 5 g per week. In this scenario, the absorption of calcium, magnesium, and potassium rarely exceeds 2–3% of the respective RDAs, indicating that algae consumption in Europe contributes only marginally to daily mineral requirements. Conversely, absorption becomes substantially more significant in populations with high habitual consumption. In South Korea, for instance, Mg absorption exceeds 30% for arame and wakame. Kombu showed the highest absorption of Na and K, reflecting its high mineral content, but this raises questions about Na exposure in frequent consumers. These results demonstrate that the intake of macronutrients is not uniformly beneficial. While Ca, Mg and K can contribute to adequate nutritional intake in regions where seaweed is consumed in large quantities, the high sodium content of some brown macroalgae could pose a dietary concern. Overall, the absorption data confirm that the nutritional relevance of macronutrients from seaweed is highly context-dependent, with species choice playing a decisive role in determining whether consumption provides significant mineral support or requires moderation. Trace element (essential, toxic and potentially toxic) absorption also showed variable rates. While elements such as Fe and Zn may contribute significantly to the diet of populations with high habitual seaweed consumption, the European scenario modeled on conservative weekly intake shows negligible nutritional impact. Conversely, As and I are the most problematic elements, with kombu consistently exceeding the recommended limits for both I and As, even in the European minimum intake scenario. The high I uptake associated with brown macroalgae confirms previous findings on their high bioaccumulation capacity. Meanwhile, As loading highlights the need for speciation analysis to distinguish between organic and inorganic forms. While Cd and Pb levels generally remain within acceptable limits, some species, particularly nori and arame, approach thresholds that require the attention of regular consumers. Overall, the intake data reinforce the need for harmonized regulatory limits for toxic elements in seaweed products and highlight the importance of species-specific consumption guidelines, especially for kombu, whose elemental profile poses a concrete risk, even at low intake levels.

### 3.6. Practical Implications

In addition to highlighting regulatory challenges and compositional differences among the analyzed species, the results of this study reveal practical implications for producers, regulatory authorities and consumers. Notably, the absence of harmonized EU-wide limits for elements, such as As and I, requires operational guidelines to ensure the safe use of seaweed in the EU food market. One key area concerns communicating with consumers. The high I concentrations observed in kombu seaweed (3125.61 ± 54.48 mg/kg), along with Cd and As levels in several species that exceed CEVA reference values, suggest the need for mandatory label warnings similar to those already required for other foods with high levels of micronutrients or contaminants. Instructions such as ‘Do not consume more than X grams per day/week’, ‘Not suitable for individuals with thyroid disorders’, or ‘Product to be consumed after rehydration and cooking’ could help to reduce the risk of excessive exposure, particularly for occasional consumers.

At the same time, the results emphasize the need for standardized testing throughout the EU supply chain. In particular, the high As content of seaweed means that analyzing only the total concentration is insufficient for an accurate risk assessment. Consequently, As speciation and I quantification following heat treatment should become mandatory analytical procedures for seaweed-based products, given that cooking can significantly reduce the levels of these elements. Adopting harmonized protocols would reduce variability among producers and provide regulatory authorities with more reliable data.

Finally, to better illustrate the impact of the study, it is useful to translate the experimental findings into realistic consumption scenarios. In the EU context, assuming an occasional intake of 5 g per week, iodine intake from kombu still reaches 1459% of the RDA, while As intake exceeds 150% of the weekly BMDL01, even without daily consumption. In a scenario reflecting Asian dietary habits, such as in Japan (4 g per day), I intake from kombu exceeds 8000% of the RDA, while As intake reaches 869% of the toxicological limit. These examples demonstrate how exposure levels can become critical even with small portion sizes, underscoring the urgent need for specific guidelines regarding seaweed species with high I and As content.

Overall, implementing the results of this study in practical measures, such as targeted labeling, mandatory analytical protocols and explicit consumption scenarios, represents an essential step towards ensuring the safe use of algae in the European market, as well as supporting the evolution of regulations towards harmonized limits based on scientific evidence.

### 3.7. Study Limitations and Future Directions

While this study provides an integrated characterization and evaluation of the mineral content, toxic elements and fatty acid composition of commercially available edible algae, the results should be interpreted in light of certain limitations.

A fundamental limitation of this study is that the analyzed samples are not fully representative. Only one commercial product per seaweed type purchased at a single point in time was considered in the study. While this approach reflects the actual availability of seaweed-based products on the EU market, it inherently limits the ability to capture intraspecific variability, which is known to be significant. Although three analytical replicates were performed, these do not account for the variability linked to production batches, harvest seasons, geographical origins or brand-specific processing practices. The geographical origins listed in Table 1 are indicative but generic, failing to capture the heterogeneity typically observed within the same species across different producers or harvest sites. Consequently, the results presented here should be interpreted as a study of the specific commercial products analyzed, rather than as definitive descriptors of all seaweeds of the same type available on the market. Future studies should include multiple batches, different brands and samples collected across different seasons and production regions to better capture intraspecific variability and strengthen the generalizability of the findings. Including additional commercial products from various suppliers would provide valuable supplementary data and enhance the reliability of regulatory and nutritional assessments.

A second limitation is the lack of information on the impact of processing methods. Commercially available seaweeds are often dried, roasted, shredded or pulverized, and these processes can significantly impact the concentrations of essential and toxic elements. For example, drying can increase the apparent mineral content due to water loss, whereas roasting can reduce the I content through volatilization or alter the speciation of As. As this study only analyzed dried products, it is impossible to determine whether the observed concentrations reflect the original composition of fresh seaweed or are partly determined by industrial processing. Future studies should, therefore, include comparative analyses of fresh, dried and processed samples to better understand the impact of technological treatments on nutritional value and toxicological risk.

The regulatory gap is another important consideration. Currently, the EU has only established limits for Cd, Pb and Hg in algae-based food supplements (EU Regulation 2023/915). This means that there are no harmonized thresholds for algae products intended for conventional food use. However, national recommendations or guidelines do exist, such as those provided by CEVA. This complicates risk assessment and consumer protection, particularly given brown algae’s natural tendency to accumulate inorganic elements, notably I and As. Ideally, a harmonized EU regulatory framework based on scientific evidence would be developed, which could include assessing inorganic As, setting species-specific limits that account for the varying accumulation patterns of brown, red, and green algae, and establishing exposure-based thresholds that take into account the variety of ways in which algae are consumed. Finally, a comparison with the existing literature confirms that most concentrations detected in this study fall within known ranges. However, it also highlights significant deviations for certain elements and species. Such discrepancies emphasize the need for extensive, geographically diverse datasets to inform future regulatory decisions and improve our understanding of the impact of environmental, ecological, and technological factors on the elemental profile of edible algae.

Overall, while this study provides a robust and integrated assessment of commercially available algae, future research should increase sample representativeness, quantify the effects of processing methods and contribute to the definition of harmonized European guidelines. These steps are crucial for ensuring safe consumption and supporting the growing use of algae in innovative food products.

## 4. Conclusions

This study provides a comprehensive characterization of the mineral profile and fatty acid composition of six commercially available edible algae. It highlights their nutritional potential and critical safety issues associated with consumption. The algae analyzed were found to be an excellent source of macronutrients. Brown macroalgae showed the highest levels of Ca, Na and Mg, while spirulina exhibited a distinctive profile rich in P. Trace element analysis confirmed the presence of nutritionally relevant concentrations of Fe, Zn, Mn and Cu, although considerable interspecies and geographical variability was observed. Furthermore, given their high I content, attention must also be paid to toxic and potentially toxic elements. The consistently high levels of total As and Cd in particular underscore the need for rigorous monitoring and regulatory harmonization. Absorption assessment further reinforces this concern: I and As intakes exceeded toxicological thresholds in all populations studied in some cases. Consequently, these results demonstrate that even occasional consumption of certain species, particularly kombu, may pose a risk to sensitive individuals. The FA profile confirms the nutritional value of algae as a source of PUFAs, particularly EPA in red macroalgae. However, the variability observed among taxa suggests that species selection is essential to maximize nutritional benefits. Overall, the results highlight the dual nature of edible algae. While they are promising functional ingredients with valuable nutritional properties, careful management is required, particularly regarding iodine and As levels, to ensure their safe consumption. In the absence of harmonized European regulations, it is essential to establish species-specific guidelines and promote As speciation analyses to protect consumers and enable the safe integration of algae into innovative food products and dietary practices.

## Figures and Tables

**Table 1:** Samples of edible seaweeds under study. In the table, their classification, scientific name and geographical origin are also reported.

Seaweed	Type	Species	Geographical Origin
Dulse	Red macroalgae	*Palmaria palmata*	UE
Nori		*Porphyra yezoensis*	Japan
Wakame	Brown macroalgae	*Undaria pinnatifida*	UE (Atlantic Ocean)
Arame		*Eisenia bicyclis*	Japan
Kombu		*Laminaria ochroleuca*	UE (Atlantic Ocean)
Spirulina	Blue–green microalgae	*Arthrospira platensis*	No-UE

**Table 2:** Concentrations (mg/kg, dry weight) of macro-elements in edible seaweeds. The results are reported as the mean ± standard deviation of triplicate analyses for each sample. Bold *p*-values are statistically significant according to the one-way ANOVA (*p* < 0.05). ^a–f^: Different superscript letters in the same column indicate significantly different values for a given parameter (*p* < 0.05 according to the post hoc Tukey’s HSD test). The same superscript letters in the same column indicate that the values are not significantly different (*p* > 0.05 according to the post hoc Tukey’s HSD test).

Species	Ca	K	Na	Mg	P
Dulse	2910.51 ± 23.23 ^a^	30,479.50 ± 200.47 ^a^	1987.46 ± 56.14 ^a^	5427.85 ± 89.50 ^a^	2819.34 ± 35.46 ^a^
Wakame	9205.65 ± 67.66 ^b^	3949.72 ± 93.99 ^b^	20,900.37 ± 113.76 ^b^	13,356.84 ± 82.38 ^b^	3257.13 ± 67.91 ^b^
Kombu	10,253.60 ± 51.60 ^c^	46,360.12 ± 548.84 ^c^	49,419.96 ± 1035.35 ^c^	10,615.07 ± 205.42 ^c^	1885.47 ± 28.96 ^c,d^
Arame	6950.30 ± 66.50 ^d^	20,005.36 ± 143.38 ^d^	10,837.92 ± 79.60 ^d^	17,496.40 ± 157.80 ^d^	1909.30 ± 13.43 ^d,c^
Nori	5570.39 ± 96.77 ^e^	25,573.49 ± 262.16 ^e^	3946.62 ± 81.92 ^e^	3151.35 ± 37.24 ^e^	2798.71 ± 61.47 ^a^
Spirulina	1516.95 ± 51.82 ^f^	16,538.43 ± 213.47 ^f^	6198.99 ± 120.26 ^f^	2580.72 ± 112.12 ^f^	8912.58 ± 117.20 ^e^
*p*-value	**<0.05**	**<0.05**	**<0.05**	**<0.05**	**<0.05**
Red macroalgae	4240.45 ± 1880.82	28,026.49 ± 3469.07	2967.04 ± 1385.34	4289.60 ± 1609.73	2809.02 ± 14.59
Brown macroalgae	8803.18 ± 1688.03	23,438.40 ± 21,412.61	27,052.75 ± 20,013.30	13,822.77 ± 3464.25	2350.63 ± 785.14
Blue–green microalgae	1516.95 ± 51.82	16,538.43 ± 213.46	6198.99 ± 120.26	2580.72 ± 112.12	8912.58 ± 117.20

**Table 3:** Trace element concentrations (mg/kg, dry weight) in the analyzed edible seaweeds. The results are reported as the mean ± standard deviation of triplicate analyses for each sample. Bold *p*-values are statistically significant according to the one-way ANOVA (*p* < 0.05). a–f: Different superscript letters in the same column indicate significantly different values for a given parameter (*p* < 0.05 according to the post hoc Tukey’s HSD test). The same superscript letters in the same column indicate that the values are not significantly different (*p* > 0.05 according to the post hoc Tukey’s HSD test). Values in green indicate that the limit has been exceeded.

Species	Fe	Zn	Cu	Se	Mn	Cr	Sr	Mo	I	Co
Dulse	407.07 ± 5.62 ^a^	30.02 ± 1.92 ^a^	4.51 ± 0.55 ^a^	0.19 ± 0.02 ^a^	24.34 ± 1.76 ^a^	1.90 ± 0.07 ^a^	25.82 ± 1.94 ^a^	0.33 ± 0.02 ^a^	187.79 ± 2.30 ^a^	0.38 ± 0.03 ^a^
Wakame	159.30 ± 7.55 ^b^	37.66 ± 1.45 ^b,e^	2.50 ± 0.13 ^b,c,e^	0.68 ± 0.04 ^b,d^	7.03 ± 0.56 ^b^	1.56 ± 0.12 ^b^	799.71 ± 3.93 ^b^	0.09 ± 0.02 ^b,c^	207.74 ± 2.31 ^a^	0.13 ± 0.01 ^b,f^
Kombu	301.64 ± 3.35 ^c^	22.57 ± 1.51 ^c,d^	2.11 ± 0.13 ^c,b^	0.35 ± 0.04 ^c^	17.22 ± 1.66 ^c,e^	2.73 ± 0.10 ^c^	334.84 ± 10.99 ^c^	0.13 ± 0.02 ^c,b^	**3125.61 ± 54.48** ^b^	0.06 ± 0.01 ^c^
Arame	255.25 ± 6.63 ^d^	19.14 ± 1.15 ^d,e^	5.04 ± 0.29 ^a^	0.70 ± 0.03 ^d,b^	33.14 ± 1.61 ^d^	2.02 ± 0.07 ^a^	66.33 ± 4.33 ^d^	0.22 ± 0.02 ^d^	79.22 ± 3.01 ^c,d^	0.45 ± 0.02 ^d^
Nori	130.25 ± 3.53 ^e^	38.89 ± 1.45 ^e,b^	7.92 ± 0.19 ^d^	0.53 ± 0.02 ^e^	18.93 ± 1.05 ^e,c^	0.57 ± 0.07 ^d^	33.68 ± 2.89 ^a,e^	0.54 ± 0.04 ^e^	37.40 ± 1.64 ^d,c,e^	0.23 ± 0.02 ^e,f^
Spirulina	554.96 ± 4.17 ^f^	14.08 ± 1.30 ^f^	3.16 ± 0.16 ^e,b^	0.96 ± 0.06 ^f^	25.34 ± 0.46 ^a^	2.03 ± 0.08 ^a^	46.03 ± 2.82 ^e,a^	0.36 ± 0.03 ^a^	10.56 ± 0.95 ^e,d^	0.18 ± 0.03 ^f,b,e^
*p*-value	**<0.05**	**<0.05**	**<0.05**	**<0.05**	**<0.05**	**<0.05**	**<0.05**	**<0.05**	**<0.05**	**<0.05**
Red macroalgae	268.66 ± 195.75	34.46 ± 6.27	6.22 ± 2.41	0.36 ± 0.24	21.63 ± 3.83	1.24 ± 0.94	29.75 ± 5.56	0.44 ± 0.15	112.60 ± 106.34	0.31 ± 0.10
Brown macroalgae	238.73 ± 72.59	26.46 ± 9.85	3.22 ± 1.59	0.58 ± 0.20	19.13 ± 13.16	2.10 ± 0.59	400.29 ± 371.05	0.14 ± 0.07	1137.52 ± 1722.93	0.21 ± 0.21
Blue–green microalgae	554.96 ± 4.17	14.08 ± 1.30	3.16 ± 0.16	0.96 ± 0.06	25.34 ± 0.46	2.03 ± 0.08	46.03 ± 2.82	0.36 ± 0.03	10.56 ± 0.95	0.18 ± 0.03

**Table 4:** Concentration of Cd, Pb, As, Hg, Ni, and Sn (mg/kg, dry weight) in the analyzed seaweeds. The results are reported as the mean ± standard deviation. Bold *p*-values are statistically significant according to the one-way ANOVA (*p* < 0.05). a–f: Different superscript letters in the same column indicate significantly different values for a given parameter (*p* < 0.05 according to the post hoc Tukey’s HSD test). The same superscript letters in the same column indicate that the values are not significantly different (*p* > 0.05 according to the post hoc Tukey’s HSD test). Values in green indicate that the limit has been exceeded.

Species	Cd	Pb	As	Hg	Ni	Sn	Sb
Dulse	**0.54 ± 0.05** ^a^	0.24 ± 0.02 ^a^	**15.81 ± 0.44** ^a^	0.01 ± 0.00 ^a^	4.01 ± 0.11 ^a^	0.03 ± 0.00 ^a^	0.06 ± 0.01 ^a^
Wakame	**0.78 ± 0.04** ^b^	0.33 ± 0.02 ^b^	**13.86 ± 0.89** ^a,d^	0.01 ± 0.00 ^a^	0.98 ± 0.07 ^b^	0.02 ± 0.00 ^a,d^	0.04 ± 0.00 ^b,e^
Kombu	**0.51 ± 0.03** ^a^	0.13 ± 0.01 ^c,e,f^	**45.63 ± 3.33** ^b^	0.03 ± 0.01 ^b^	0.68 ± 0.07 ^c,e,f^	0.05 ± 0.01 ^b,e^	0.02 ± 0.00 ^c,e^
Arame	0.25 ± 0.04 ^c,e^	0.82 ± 0.02 ^d^	**7.03 ± 0.44** ^c,d,e^	0.02 ± 0.00 ^c,d^	3.43 ± 0.08 ^d^	0.11 ± 0.01 ^c^	0.08 ± 0.01 ^d^
Nori	**1.06 ± 0.07** ^d^	0.10 ± 0.02 ^e,c,f^	**10.81 ± 0.44** ^d,a,c^	0.01 ± 0.00 ^a^	0.53 ± 0.08 ^e,c,f^	0.01 ± 0.00 ^d,a^	0.05 ± 0.01 ^a^
Spirulina	0.22 ± 0.03 ^e,c^	0.13 ± 0.01 ^f,c,e^	**4.92 ± 0.39** ^e,c^	0.02 ± 0.00 ^d,c^	0.51 ± 0.05 ^f,c,e^	0.05 ± 0.01 ^e,b^	0.03 ± 0.00 ^e,b,c^
*p*-value	**<0.05**	**<0.05**	**<0.05**	**<0.05**	**<0.05**	**<0.05**	**<0.05**
Red macroalgae	0.80 ± 0.37	0.17 ± 0.10	13.31 ± 3.53	0.01 ± 0.00	2.27 ± 2.46	0.02 ± 0.01	0.06 ± 0.00
Brown macroalgae	0.51 ± 0.27	0.43 ± 0.36	22.17 ± 20.60	0.02 ± 0.01	1.70 ± 1.51	0.06 ±0.05	0.05 ± 0.03
Blue–green microalgae	0.22 ± 0.03	0.13 ± 0.01	4.92 ± 0.39	0.02 ± 0.00	0.51 ± 0.05	0.05 ± 0.01	0.03 ± 0.00

**Table 5:** The limits set by French agency CEVA for certain inorganic contaminants.

Element	mg/kg on a Dry Weight Basis
Inorganic Arsenic (iAs)	3
Cadmium (Cd)	0.5
Mercury (Hg)	0.1
Lead (Pb)	5
Tin (Sn)	5
Iodine (I)	2000

**Table 6:** FA composition (g/100 g lipid extract) and AI and TI values of the seaweed samples. The results are expressed as the mean ± standard deviation of triplicate analyses conducted for every sample. Bold *p*-values are statistically significant according to the one-way ANOVA (*p* < 0.05). a–f: Different superscript letters in the same column indicate significantly different values for a given parameter (*p* < 0.05 according to the post hoc Tukey’s HSD test). The same superscript column in the same row indicates that the values are not significantly different (*p* > 0.05 according to the post hoc Tukey’s HSD test).

	Dulse	Wakame	Kombu	Arame	Nori	Spirulina	*p*-Value	Red Macroalgae	Brown Macroalgae	Blue–Green Microalgae
C12:0	0.19 ± 0.00 ^a^	0.18 ± 0.01 ^a^	0.13 ± 0.01 ^b^	0.03 ± 0.00 ^c^	0.17 ± 0.01 ^d^	0.01 ± 0.00 ^e^	**<0.05**	0.18 ± 0.01	0.11 ± 0.08	0.01 ± 0.00
C14:0	3.06 ± 0.10 ^a^	3.90 ± 0.15 ^b^	3.26 ± 0.12 ^a^	4.52 ± 0.21 ^c^	0.38 ± 0.02 ^d,e^	0.48 ± 0.01 ^e,d^	**<0.05**	1.72 ± 1.90	3.89 ± 0.63	0.48 ± 0.01
C15:0	0.34 ± 0.02 ^a^	0.36 ±0.02 ^a^	0.24 ± 0.01 ^b^	0.34 ± 0.02 ^a^	0.13 ± 0.01 ^c^	0.03 ± 0.00 ^d^	**<0.05**	0.24 ± 0.15	0.31 ± 0.06	0.03 ± 0.00
C16:0	25.17 ± 0.87 ^a^	28.40 ± 0.96 ^b,a,d^	32.10 ± 1.35 ^c,d,e^	25.54 ± 0.90 ^a,b^	30.04 ± 1.05 ^d,b,c^	33.35 ± 1.24 ^e,c^	**<0.05**	27.61 ± 3.44	28.68 ± 3.29	33.35 ± 1.24
C17:0	0.32 ± 0.02 ^a^	0.25 ± 0.02 ^b,c,d^	0.28 ± 0.01 ^c,b^	0.25 ± 0.01 ^d,b^	0.06 ± 0.00 ^e^	0.13 ± 0.01 ^f^	**<0.05**	0.19 ± 0.18	0.26 ± 0.02	0.13 ± 0.01
C18:0	5.14 ± 0.24 ^a^	2.82 ± 0.15 ^b,d^	1.94 ± 0.72 ^c,d,e^	2.66 ± 0.12 ^d,b,c^	1.19 ± 0.05 ^e,c^	6.64 ± 0.03 ^f^	**<0.05**	3.17 ± 2.79	2.47 ± 0.47	6.64 ± 0.03
C20:0	0.13 ± 0.01 ^a^	0.61 ± 0.03 ^b^	2.36 ± 0.08 ^c^	1.42 ± 0.05 ^d^	0.03 ± 0.00 ^a^	0.07 ± 0.00 ^a^	**<0.05**	0.08 ± 0.07	1.46 ± 0.88	0.07 ± 0.00
C22:0	0.27 ± 0.06 ^a^	0.49 ± 0.02 ^b^	0.32 ± 0.02 ^a^	0.23 ± 0.01 ^a^	0.33 ± 0.02 ^a^	0.13 ± 0.01 ^c^	**<0.05**	0.30 ± 0.04	0.35 ± 0.13	0.13 ± 0.01
C24:0	0.23 ± 0.03 ^a^	2.55 ± 0.13 ^b^	3.62 ± 0.11 ^c^	0.27 ± 0.01 ^a^	0.20 ± 0.01 ^a,d^	0.03 ± 0.00 ^d,a^	**<0.05**	0.22 ± 0.02	2.15 ± 1.71	0.03 ± 0.00
∑ SFA	34.85 ± 0.80	39.56 ± 0.82	44.25 ± 1.22	35.26 ± 0.82	32.53 ± 0.85	40.87 ± 1.12		33.69 ± 1.64	39.69 ± 4.50	40.87 ± 1.12
C16:1 n-9	0.57 ± 0.03 ^a^	0.17 ±0.01 ^b,c,d^	0.20 ± 0.01 ^c,b,d^	0.23 ± 0.02 ^d,b,c^	0.50 ± 0.03 ^a^	0.67 ±0.04 ^e^	**<0.05**	0.54 ± 0.05	0.20 ± 0.03	0.67 ±0.04
C16:1 n-7	0.40 ± 0.02 ^a^	5.24 ± 0.35 ^b,c^	0.21 ± 0.01 ^a^	5.33 ± 0.41 ^c,b^	0.42 ± 0.02 ^a^	1.17 ± 0.07 ^d^	**<0.05**	0.41 ± 0.01	3.59 ± 2.93	1.17 ± 0.07
C17:1	0.82 ± 0.03 ^a^	1.53 ± 0.06 ^b^	0.87 ± 0.05 ^a^	0.21 ± 0.01 ^c,e^	0.35 ± 0.02 ^d,e^	0.30 ± 0.02 ^e,c,d^	**<0.05**	0.59 ± 0.33	0.87 ± 0.66	0.30 ± 0.02
C18:1 n-9	17.31 ± 0.65 ^a^	18.05 ± 0.80 ^a^	15.37 ± 0.66 ^b,a^	27.68 ± 1.02 ^c^	15.54 ± 0.46 ^a,b^	20.68 ± 0.18 ^d^	**<0.05**	16.43 ± 1.25	20.37 ± 6.47	20.68 ± 0.18
C18:1 n-7	0.70 ± 0.04 ^a^	0.56 ± 0.02 ^b,c^	0.78 ± 0.06 ^a^	0.58 ± 0.03 ^c,b^	0.72 ± 0.05 ^a^	0.41 ± 0.02 ^d^	**<0.05**	0.71 ± 0.01	0.64 ± 0.12	0.41 ± 0.02
C20:1 n-9	0.34 ± 0.02 ^a^	0.16 ± 0.01 ^b,c,d,f^	0.10 ± 0.00 ^c,b,d,f^	0.06 ± 0.00 ^d,b,c,f^	3.38 ± 0.14 ^e^	0.03 ± 0.00 ^f,b,c,d^	**<0.05**	1.86 ± 2.15	0.11 ± 0.05	0.03 ± 0.00
C22:1 n-9	0.35 ± 0.02 ^a^	0.22 ± 0.02 ^b,c,d^	0.18 ± 0.01 ^c,b,d^	0.20 ± 0.01 ^d,b,c^	0.32 ± 0.02 ^a^	0.10 ± 0.01 ^e^	**<0.05**	0.34 ± 0.02	0.20 ± 0.02	0.10 ± 0.01
∑ MUFA	20.49 ± 0.54	25.93 ± 0.64	17.71 ± 0.53	34.29 ± 0.98	21.23 ± 0.34	23.36 ± 0.14		20.86 ± 0.52	25.98 ± 8.29	23.36 ± 0.14
C18:2 n-6	4.49 ± 0.05 ^a^	7.24 ± 0.40 ^b,c,d^	7.92 ± 0.51 ^c,b,d^	7.72 ± 0.44 ^d,b,c^	3.44 ± 0.19 ^a^	19.05 ± 0.76 ^e^	**<0.05**	3.97 ± 0.74	7.63 ± 0.35	19.05 ± 0.76
C18:3 n-6	3.94 ± 0.10 ^a^	0.91 ± 0.05 ^b,c,d^	0.77 ± 0.03 ^c,b,d,e^	1.00 ± 0.04 ^d,b,c^	0.37 ± 0.02 ^e,c^	8.81 ± 0.45 ^f^	**<0.05**	2.16 ± 2.52	0.89 ± 0.12	8.81 ± 0.45
C18:3 n-3	0.10 ± 0.00 ^a^	4.33 ± 0.21 ^b,c^	4.05 ± 0.27 ^c,b^	3.16 ± 0.17 ^d^	0.32 ± 0.02 ^a^	0.10 ± 0.01 ^a^	**<0.05**	0.21 ± 0.16	3.85 ± 0.61	0.10 ± 0.01
C20:2 n-6	0.19 ± 0.01 ^a^	0.09 ± 0.00 ^b,c,a,e^	0.11 ± 0.01 ^c,b,a,e^	0.14 ± 0.01 ^a,b,c,e^	0.95 ± 0.05 ^d^	0.11 ± 0.01 ^e,b,c,a^	**<0.05**	0.57 ± 0.54	0.11 ± 0.03	0.11 ± 0.01
C20:3 n-6	1.73 ± 0.06 ^a^	1.09 ± 0.05 ^b^	0.73 ± 0.04 ^c^	0.54 ± 0.02 ^d^	1.88 ± 0.05 ^e^	0.25 ± 0.02 ^f^	**<0.05**	1.81 ± 0.11	0.79 ± 0.28	0.25 ± 0.02
C20:4 n-6	3.41 ± 0.17 ^a^	13.32 ± 0.75 ^b,c,d^	12.89 ± 0.60 ^c,b,d^	12.23 ± 0.58 ^d,b,c^	3.32 ± 0.21 ^a^	0.06 ± 0.00 ^e^	**<0.05**	3.37 ± 0.06	12.81 ± 0.55	0.06 ± 0.00
C20:3 n-3	0.05 ± 0.01	0.05 ± 0.00	0.05 ± 0.00	0.05 ± 0.00	0.06 ± 0.01	0.05 ± 0.00	>0.05	0.06 ± 0.01	0.04 ± 0.01	0.01 ± 0.00
C20:4 n-3	0.60 ± 0.04 ^a^	0.55 ± 0.02 ^a^	0.60 ± 0.03 ^a^	0.58 ± 0.02 ^a^	0.61 ± 0.03 ^a^	0.08 ± 0.00 ^b^	**<0.05**	0.61 ± 0.01	0.58 ± 0.03	0.08 ± 0.00
C20:5 n-3	27.21 ± 0.01 ^a^	5.81 ± 0.18 ^b^	8.45 ± 0.32 ^c^	3.44 ± 0.10 ^d,f^	33.65 ± 1.54 ^e^	2.04 ± 0.05 ^f,d^	**<0.05**	30.43 ± 4.55	5.90 ± 2.51	2.04 ± 0.05
C22:6 n-3	0.05 ± 0.00 ^a^	0.04 ± 0.00 ^a^	0.05 ± 0.00 ^a^	0.07 ± 0.00 ^a^	0.06 ± 0.00 ^a^	3.01 ± 0.03 ^b^	**<0.05**	0.06 ± 0.01	0.05 ± 0.02	3.01 ± 0.03
∑ PUFA	41.77 ± 0.10	33.42 ± 0.60	35.61 ± 0.44	28.93 ± 0.46	44.66 ± 1.16	33.52 ± 0.40		43.22 ± 2.04	32.65 ± 3.41	33.52 ± 0.40
AI	0.60	0.76	0.85	0.69	0.48	0.62	-	0.54	0.77	0.62
TI	0.32	0.62	0.62	0.65	0.26	0.97	-	0.29	0.63	0.97
ω-3/ω-6	2.06	0.48	0.59	0.34	3.48	0.19	-	-	-	-

**Table 7:** Uptake percentage (%) of mineral elements from the consumption of seaweed samples in relation to different population habits. Any percentages above 100 are displayed in red.

Uptake (%)	Population	Dulse	Wakame	Kombu	Arame	Nori	Spirulina
Ca	Chinese	2	6	7	5	4	1
	Japanese	2	5	5	4	3	1
	South Korean	3	10	11	7	6	2
	European	<1	1	1	1	1	<1
K	Chinese	8	1	12	5	7	4
	Japanese	6	1	9	4	5	3
	South Korean	13	2	20	9	11	7
	European	1	<1	2	1	1	1
Na	Chinese	1	7	17	4	1	2
	Japanese	1	6	13	3	1	2
	South Korean	1	12	28	6	2	4
	European	<1	1	2	1	<1	<1
Mg	Chinese	8	19	15	24	4	4
	Japanese	6	14	11	19	3	3
	South Korean	12	30	24	40	7	6
	European	1	3	2	3	1	1
Fe	Chinese	15	6	11	10	5	21
	Japanese	12	5	9	7	4	16
	South Korean	25	10	18	16	8	34
	European	2	1	2	1	1	3
Zn	Chinese	2	2	1	1	2	1
	Japanese	1	2	1	1	2	1
	South Korean	3	3	2	2	3	1
	European	<1	<1	<1	<1	<1	<1
Cu	Chinese	2	1	1	3	4	2
	Japanese	2	1	1	2	3	1
	South Korean	4	2	2	4	7	3
	European	<1	<1	<1	<1	1	<1
Se	Chinese	2	7	3	7	5	9
	Japanese	1	5	3	5	4	7
	South Korean	3	11	5	11	8	15
	European	<1	1	<1	1	1	1
Mn	Chinese	6	2	5	9	5	7
	Japanese	5	1	3	7	4	5
	South Korean	10	3	7	14	8	11
	European	1	<1	1	1	1	1
Cd	Chinese	11	16	11	5	22	5
	Japanese	9	12	8	4	17	4
	South Korean	19	26	18	8	36	7
	European	2	2	1	1	3	1
Pb	Chinese	4	5	2	12	2	2
	Japanese	3	4	2	9	1	2
	South Korean	6	8	3	20	3	3
	European	1	1	<1	2	<1	<1
As	Chinese	392	343	1130	174	268	122
	Japanese	301	264	869	134	206	94
	South Korean	694	561	1847	285	438	199
	European	53	46	152	23	36	16
Hg	Chinese	<1	<1	<1	<1	<1	<1
	Japanese	<1	<1	<1	<1	<1	<1
	South Korean	<1	<1	<1	<1	<1	<1
	European	<1	<1	<1	<1	<1	<1
P	Chinese	2	2	1	1	2	7
	Japanese	2	2	1	1	2	5
	South Korean	3	4	2	2	3	11
	European	<1	<1	<1	<1	<1	1
Cr	Chinese	25	20	36	27	8	26
	Japanese	19	16	27	20	6	20
	South Korean	40	33	58	43	12	43
	European	3	3	5	4	1	4
Mo	Chinese	3	1	1	2	6	4
	Japanese	3	1	1	2	4	3
	South Korean	6	2	2	4	9	6
	European	1	<1	<1	<1	1	1
Ni	Chinese	1	<1	<1	1	<1	<1
	Japanese	1	<1	<1	1	<1	<1
	South Korean	2	<1	<1	2	<1	<1
	European	<1	<1	<1	<1	<1	<1
Sb	Chinese	1	<1	<1	1	1	<1
	Japanese	<1	<1	<1	1	<1	<1
	South Korean	1	1	<1	1	1	<1
	European	<1	<1	<1	<1	<1	<1
I	Chinese	651	720	10,835	275	130	37
	Japanese	501	554	8335	211	100	28
	South Korean	1064	1177	17,712	449	212	60
	European	88	97	1459	37	18	5
Co	Chinese	12	4	2	15	8	6
	Japanese	9	3	1	11	6	5
	South Korean	20	7	3	24	12	10
	European	2	1	<1	2	1	1

## Data Availability

The original contributions presented in this study are included in the article/Supplementary Materials. Further inquiries can be directed to the corresponding author.

## Supplementary Materials

The following supporting information can be downloaded at https://www.mdpi.com/article/10.3390/foods15183219/s1: Table S1: Parameters and data of analytical validation resulted from the assessment of ICP-OES (target analytes: Ca, K, Na, Mg, P, Fe and Zn), ICP-MS (target analytes: Cu, Se, I, Sr, Co, Mo, Cd, Pb, As, Ni, Sn and Sb) and DMA-80 (target analyte: Hg) methods.
