# Supplementary material for: Commercial Edible Seaweeds: Characterization by Mineral Content and Fatty Acid Composition
Source: Foods. 2026 Sep 11;15(18):3219. doi: 10.3390/foods15183219 (PMC13605656; doi:10.3390/foods15183219)
Supplement: Supplementary file 1 [file foods-15-03219-s001.zip › foods-4510808-supplementary.pdf]

Supplementary Material

Commercial Edible Seaweeds: Characterization by Mineral Content and Fatty Acid Composition

Vincenzo Nava[1†], Angela Giorgia Potortì[1†], Ambrogina Albergamo[1*], Roberto Sturniolo[1,2], Irene Maria Spanò[1,2] and Giuseppa Di Bella[1]

[1] Department of Biomedical, Dental and Morphological and Functional Imaging Sciences (BIOMORF), University of Messina, Viale Palatucci 13, 98168 Messina, Italy; vnava@unime.it (V.N.); agpotorti@unime.it (A.G.P.); roberto.sturniolo@studenti.unime.it (R.S.); irene.spano@studenti.unime.it (I.M.S.); gdibella@unime.it (G.D.B.)

[2] Department of Chemical, Biological, Pharmaceutical and Environmental Sciences (CHIBIOFARAM), University of Messina, 98166 Messina, Italy

* Correspondence: aalbergamo@unime.it

† These authors contributed equally to the work.

1. Analytical validation of methods employed in the study

The validation of the three methods (ICP-MS, ICP-OES and DMA-80) used for the determination of the inorganic elements has been assessed in terms of their linearity, sensitivity, accuracy and precision. All the results obtained are shown in Table S1. To assess linearity, seven-point calibration curves were constructed using multi-element standard solutions. The linearity of these curves was verified by calculating their correlation coefficients (R^2). The standard concentration ranges were 0.5–50 mg/L, 1–100 μg/L and 1–100 μg/L for the ICP-OES, ICP-MS and DMA-80 protocols, respectively. All methods demonstrated excellent linearity for all of the analyzed elements, with the R^2 values consistently exceeding 0.9991 (Table S1).

The assessment of analytical sensitivity involved the determination of the limits of detection (LOD) and quantification (LOQ) for each element. The following formulas were used to obtain, respectively, the LOD and LOQ: $3.3\sigma/S$ and $10\sigma/S$, where σ is the standard deviation of the mean value obtained from analyzing ten blank samples and S is the slope of the corresponding calibration curve. The lowest LOD and LOQ values were obtained for Hg and Sn (0.003 and 0.010 mg/kg, respectively), whereas the highest values were obtained for K (0.755 and 2.492 mg/kg, respectively). The accuracy of the methods was determined using the certified reference matrix spinach leaves (CRM: NIST1570a), with six replicates. The accuracy was derived by deducting the experimental value from the reference value and expressing the outcome as a percentage recovery. Moreover, in case of missing analytes in the CRM, known concentrations of the relative standard solutions were spiked. Satisfactory recoveries were observed since the highest average recovery was obtained for Pb (101.20%), while the lowest was obtained for K (92.00%) (Table S1). Finally, the intra-day and the inter-day precision of the methods were calculated by analyzing the certified matrix and the spiked samples on the same day and over a longer period of one week, respectively. The results, shown as relative standard deviation (RSD%), were found to be less than 1.1% and 1.3%, respectively (see Table S1).

Table S1. Parameters and data of analytical validation resulted from the assessment of ICP-OES (target analytes: Ca, K, Na, Mg, P, Fe and Zn), ICP-MS (target analytes: Cu, Se, I, Sr, Co, Mo, Cd, Pb, As, Ni, Sn and Sb) and DMA-80 (target analyte: Hg) methods

	**Linearity (R^2)**	**LOD (mg/kg)**	**LOQ (mg/kg)**	**Recovery (%)**	**Precision (RSD%)**	
					**Intraday**	**Interday**
			**Macroelements**			
Ca	0.9993	0.701	2.313	93.75	1.0	1.2
K	0.9991	0.755	2.492	92.00	1.0	1.2
Na	0.9992	0.695	2.294	93.50	1.0	1.2
Mg	0.9995	0.601	1.983	95.86	1.1	1.2
P	0.9993	0.643	2.125	94.25	1.0	1.3
			**Trace elements**			
Fe	0.9996	0.586	1.933	95.15	0.8	0.9
Zn	0.9996	0.578	1.907	97.00	0.9	1.0
Cu	0.9994	0.022	0.073	96.83	0.8	0.9
Se	0.9994	0.015	0.050	95.44	0.9	1.0
Mn	0.9996	0.020	0.066	97.05	1.0	1.1
Cr	0.9994	0.012	0.040	96.15	0.8	1.0
I	0.9995	0.028	0.092	96.70	0.9	1.0
Sr	0.9994	0.029	0.096	95.48	1.0	1.1
Co	0.9994	0.010	0.033	95.14	0.9	1.0
Mo	0.9995	0.010	0.033	97.11	0.7	0.9
			**Toxic and potentially toxic elements**			
Cd	0.9999	0.004	0.013	100.22	1.0	1.1
Pb	0.9999	0.004	0.013	101.20	0.8	0.9
As	0.9997	0.010	0.033	98.35	1.0	1.1
Hg	0.9998	0.003	0.010	97.00	0.9	1.0
Ni	0.9997	0.012	0.040	97.54	0.8	0.9
Sn	0.9995	0.003	0.010	96.45	1.0	1.2
Sb	0.9994	0.004	0.013	97.65	1.1	1.2
